# Supplementary material for: Histone Chaperones and Digestive Cancer: A Review of the Literature
Source: Cancers (Basel). 2022 Nov 14;14(22):5584. doi: 10.3390/cancers14225584 (PMC9688693; doi:10.3390/cancers14225584)
Supplement: Supplementary file 1 [file cancers-14-05584-s001.zip › Supplementary Method S1.pdf]

1. Search strategy

1.1 Pubmed search strategy

1) ((((("Esophageal Neoplasms"[Mesh]) OR "Stomach Neoplasms"[Mesh]) OR "Liver Neoplasms"[Mesh]) OR "Pancreatic Neoplasms"[Mesh]) OR "Colorectal Neoplasms"[Mesh]) OR "Gastrointestinal Neoplasms"[Mesh] 657,819
2) Histone chaperone[Title/Abstract]...995
3) ((Anti-silencing function 1[Title/Abstract]) OR (ASF1A[Title/Abstract])) OR (ASF1B[Title/Abstract]) 175
4) (((((Chromatin assembly factor 1[Title/Abstract]) OR (CHAF1A[Title/Abstract])) OR (CHAF1B[Title/Abstract])) OR (CAF1[Title/Abstract])) OR (Chromatin Assembly Factor 1 Subunit A[Title/Abstract])) OR (Chromatin Assembly Factor 1 Subunit B[Title/Abstract]) 770
5) ((((FACT complex[Title/Abstract]) OR (Suppressor of Ty 16[Title/Abstract])) OR (SPT16[Title/Abstract])) OR (Structure-specific recognition protein 1[Title/Abstract])) OR (SSRP1[Title/Abstract]) 344
6) (((Death domain-associated protein 6[Title/Abstract]) OR (DAXX[Title/Abstract])) OR (Alpha-thalassaemia/mental retardation syndrome X-linked[Title/Abstract])) OR (ATRX[Title/Abstract])...1913
7) (Minichromosome maintenance protein 2[Title/Abstract]) OR (MCM2[Title/Abstract]) 1382
8) TONSL[Title/Abstract] 25
9) (Retinoblastoma associated protein 46[Title/Abstract]) OR (RBAP46[Title/Abstract]) ...86
10) ((Somatic nuclear autoantigenic sperm protein[Title/Abstract]) OR (sNASP[Title/Abstract])) OR (NASP[Title/Abstract]) 145
11) (Importin 4[Title/Abstract]) OR (IPO4[Title/Abstract]) 27
12) (((Suppressor of Ty 2[Title/Abstract]) OR (Suppressor of Ty 6[Title/Abstract])) OR (SPT2[Title/Abstract])) OR (SPT6[Title/Abstract])..245
13) (Acidic-leucine-rich nuclear phosphoprotein 32E[Title/Abstract]) OR (ANP32E[Title/Abstract])

    i. 50

14) (Vacuolar protein sorting 72[Title/Abstract]) OR (VPS72[Title/Abstract]) 6
15) (Holliday junction recognition protein[Title/Abstract]) OR (HJURP[Title/Abstract])...143
16) ((Nucleosome assembly protein 1-like[Title/Abstract]) OR (NAP1L1[Title/Abstract])) OR (NAP1[Title/Abstract]) 922
17) (((((Nucleophosmin[Title/Abstract]) OR (NPM1[Title/Abstract])) OR (Nucleoplasmin 2[Title/Abstract])) OR (NPM2[Title/Abstract])) OR (Nucleoplasmin 3[Title/Abstract])) OR (NPM3[Title/Abstract])..3258
18) Nucleolin[Title/Abstract] 1608
19) (Retinoblastoma associated protein 48[Title/Abstract]) OR (RBAP48[Title/Abstract]) .117
20) (Histone regulation A[Title/Abstract]) OR (HIRA[Title/Abstract]) 655
21) (Ubinuclein 1[Title/Abstract]) OR (UBN1[Title/Abstract]) 30
22) (Calcineurin-binding protein cabin-1[Title/Abstract]) OR (CABIN1[Title/Abstract]) 80
23) Patient SE translocation[Title/Abstract] 3

24) 2 OR 3 OR 4 OR… OR 22 OR 23 12571
25) 1 AND 24 477

1.2 Ovid Embase/MEDLINE search strategy

1) esophageal neoplasm.mp. or *esophagus tumor/ 10590
2) gastric cancer.mp. or *stomach cancer/ 110932
3) hepatocellular carcinoma.mp. or *liver cell carcinoma/ 169779
4) pancreatic neoplasm.mp. or *pancreas tumor/ 17611
5) colorectal neoplasm.mp. or *colorectal tumor/ 17203
6) 1 OR 2 OR 3 OR 4 OR 5 323951
7) Anti-silencing function 1.ab,ti. OR ASF1A.ab,ti. OR ASF1B.ab,ti. 191
8) Chromatin assembly factor 1.ab,ti. OR CHAF1A.ab,ti. OR CHAF1B.ab,ti. OR CAF1.ab,ti. 725
9) FACT complex.ab,ti. OR Suppressor of Ty 16.ab,ti. OR SPT16.ab,ti. OR Structure-specific recognition protein 1.ab,ti. OR SSRP1.ab,ti. 388
10) Death domain-associated protein 6.ab,ti. OR DAXX.ab,ti. OR "Alpha-thalassaemia/mental retardation syndrome X-linked".ab,ti. OR ATRX.ab,ti. 3131
11) Minichromosome maintenance protein 2.ab,ti. OR MCM2.ab,ti.
12) TONSL.ab,ti. 30
13) Retinoblastoma associated protein 46.ab,ti. OR RBAP46.ab,ti. 108
14) Somatic nuclear autoantigenic sperm protein.ab,ti. OR sNASP.ab,ti. OR NASP.ab,ti. .. 171
15) IPO4.ab,ti. OR Importin 4.ab,ti. 28
16) Suppressor of Ty 2.ab,ti. OR Suppressor of Ty 6.ab,ti. OR SPT2.ab,ti. OR SPT6.ab,ti. ... 262
17) Acidic-leucine-rich nuclear phosphoprotein 32E.ab,ti. OR ANP32E.ab,ti. 66
18) Vacuolar protein sorting 72.ab,ti. OR VPS72.ab,ti. 15
19) Holliday junction recognition protein.ab,ti. OR HJURP.ab,ti. 194
20) Nucleosome assembly protein 1-like.ab,ti. OR NAP1L1.ab,ti. OR NAP1.ab,ti. 1024
21) Nucleophosmin.ab,ti. OR NPM1.ab,ti. OR Nucleoplasmin 2.ab,ti. OR NPM2.ab,ti. OR Nucleoplasmin 3.ab,ti. OR NPM3.ab,ti. 7489
22) Nucleolin.ab,ti. 1947
23) Retinoblastoma associated protein 48.ab,ti. OR RBAP48.ab,ti. 133
24) Histone regulation A.ab,ti. OR HIRA.ab,ti. 937
25) Ubinuclein 1.ab,ti. OR UBN1.ab,ti. 33
26) Calcineurin-binding protein cabin-1.ab,ti. OR CABIN1.ab,ti. 82
27) Patient SE translocation.ab,ti 1
28) 7 OR 8 OR 9 OR … OR 26 OR 27 18116
29) 6 AND 28 504

2. Details of literature search

2.1 Search strategy

The PubMed, Embase, and MEDLINE databases were searched to identify studies on histone chaperones and digestive cancer from inception until June 2022, following the PRISMA (Preferred Reporting Items for Systematic Reviews and Meta-Analyses) guidelines (1). PRISMA-S Checklist is presented in Supplementary Method S1. The references of included articles were also searched to identify other eligible literature.

2.2 Eligibility criteria

The following criteria were applied to identify eligible articles for inclusion in this review: (1) articles involving histone chaperone subjects, (2) digestive cancers subjects, and (3) subjects of gene expression, prognostic value, phenotype (in vivo or in vitro), and molecular mechanism. Articles were excluded due to any of the following reasons: (1) articles with only bioinformatics analysis; (2) articles being a case report, review, comment/letter to the editor, or conference; (3) articles not written in English.

2.3 Data extraction

The research team includes 4 surgeons of general surgery and an epigenetics researcher. The data were extracted using a self-designed checklist by two review authors (Zhou Zhao and Zhao-Lun Cai) independently. The differences between the two authors were solved via discussion. When there was no agreement, the decision was solved by review with the third author (Bo Zhang). The first author, publication year, genes involved, tumors involved, role of the genes in the tumor, relative expression of the genes in the tumor, relationship of the genes with overall survival (OS) and recurrence-free survival (RFS), genes in vitro and in vivo phenotypes, and upstream and downstream mechanisms were extracted.

3. Results of study selection

Out of the 1111 articles retrieved, 404 studies were removed due to duplication through the EndNote citation manager. Then, 459 studies were excluded after the title and abstract screening. The full publications of 248 articles were checked in detail for the presence of the outcome variable and 168 studies were removed. The remaining 80 eligible studies were included in this review. After a reference search of the included articles, an additional 23 articles were included in this review. The overall study selection process is represented by a flow diagram in Figure 1.

References

1. Rethlefsen, M.L.; Kirtley, S.; Waffenschmidt, S.; Ayala, A.P.; Moher, D.; Page, M.J.; Koffel, J.B. PRISMA-S: an extension to the PRISMA Statement for Reporting Literature Searches in Systematic Reviews. Syst Rev 2021, 10, 39, doi:10.1186/s13643-020-01542-z.
