# Supplementary material for: Histone Chaperones and Digestive Cancer: A Review of the Literature
Source: Cancers (Basel). 2022 Nov 14;14(22):5584. doi: 10.3390/cancers14225584 (PMC9688693; doi:10.3390/cancers14225584)
Supplement: Supplementary file 1 [file cancers-14-05584-s001.zip › Supplementary Tables.pdf]

cancers MDPI

Table S1. Details of included histone chaperones in human.

Histone chaperone	Histone preference	Complex (or complexes)	Related Tumors
ASF1A	H3.1–, H3.2–, H3.3–H4	Multiple	HCC, GIC
ASF1B	H3.1–, H3.2–, H3.3–H4	Multiple	HCC, GC, PC
MCM2	CENP-A–, H3.1–, H3.2–, H3.3–H4	MCM2–7 complex	HCC, EC, GC, CRC
TONSL	H3–H4	MMS22L–TONSL complex	GC
RBAP46	H3–H4	HAT, HDAC, NuRF, NuRD, PRC2	NR
sNASP	H3.1–, H3.2–, H3.3–H4, H1	HAT	HCC
IPO4	H3.1–, H3.2–, H3.3–H4	Nuclear import receptor	GC
SPT2	H3–H4	ND	CRC
SPT6	H3–H4	ND	CRC
ANP32E	H2A.Z–H2B	P400–TIP60	GC, PC
HJURP	CENP-A–H4 (Cse4-H4)	Centromere assembly	HCC, PC, CRC
SET	H3–H4	INHAT, Vps75–Rtt109	NR
NAP1L1	H2A–, H2A.Z–H2B, H3–H4, H1	Nuclear import importin 9, Kap114	HCC, CRC
NPM1	H3–H4, CENP-A–H4, H1	SWAP	HCC, GC, PC, CRC, EC
Nucleolin	H2A–H2B, H1	SWAP	HCC, GC, PC, CRC, EC
CHAF1A	H3.1–H4	CAF1	HCC, GC
CHAF1B	H3.1–H4	CAF1	HCC

Histone chaperone	Histone preference	Complex (or complexes)	Related Tumors
RBAP48	H3.1–, H3.2–, H3.3–H4	CAF1, HDAC, NuRF, NuRD, and PRC2	GC
HIRA	ND	HIRA/HIR	NR
UBN1	H3.3–H4	HIRA/HIR	NR
CABIN1	ND	HIRA/HIR	NR
SPT16	H2A–H2B, H3–H4	FACT	HCC
SSRP1	H2A–H2B, H3–H4	FACT	HCC, GC, CRC
DAXX	H3.3–H4	DAXX–ATRX	GC, CRC
ATRX	ND	DAXX–ATRX	CRC, PC
VPS72	H2A.Z-H2B	SRCAP/SWR-C, P400-TIP60	HCC

ANP32E: Acidic-leucine-rich nuclear phosphoprotein 32E; ASF1A: Anti-Silencing Function 1A; ASF1B: Anti-Silencing Function 1B; ATRX: Alpha-thalassaemia/mental retardation syndrome X-linked; CABIN: Calcineurin-binding protein cabin-1; CHAF1A: Chromatin Assembly Factor 1 Subunit A; CHAF1B: Chromatin Assembly Factor 1 Subunit B; CRC: colorectal cancer; DAXX: Death domain-associated protein 6; EC: esophageal cancer; GC: gastric cancer; HATs: Histone acetyltransferases; HCC: hepatocellular carcinoma; HDAC: Histone deacetylase; HIRA: Histone regulation A; HJURP: Holliday Junction Recognition Protein; hTERT: Human telomerase reverse transcriptase; INHAT: Inhibitor of histone acetyltransferases; IPO4: Importin 4; MCM2: Minichromosome maintenance protein 2; MMS22L: Methyl Methanesulfonate-Sensitivity Protein 22-Like; NAP1L1: Nucleosome Assembly Protein 1 Like 1; ND, not defined; NPM1: Nucleophosmin 1; NR: not reported; NuRF: Nucleosome Remodeling Factor; PC: pancreatic cancer; RBAP46: Retinoblastoma associated protein 46; RBAP48: Retinoblastoma associated protein 48; SET: Patient SE translocation; PRC2: Polycomb repressive complex 2; sNASP: Somatic nuclear autoantigenic sperm protein; SPT2: Suppressor of Ty 2; SPT16: Suppressor Of Ty 16; SSRP1: Structure Specific Recognition Protein 1. UBN1: Ubinuclein 1; VPS72: protein sorting 72 homologue; TONSL: Tonsuku Like.

Table S2. Included literature on selected histone chaperones in digestive cancer.

ID	Histone chaperone	First author	Year	Tumor	Reference number
1	ANP32E	Zhu	2022	GC	[1]
2		Zhang	2020	PDAC	[2]
3		Ma	2021	PDAC	[3]
4	ASF1A	Liang	2017	CRC	[4]
5		Qiu	2019	CRC	[5]
6		Wu	2019	HCC	[6]
7		Chen	2022	GC	[7]
8		Zhang	2022	HCC	[8]
9		Ouyang	2021	HCC	[9]
10		Zhan	2021	HCC	[10]
11		Kim	2022	PDAC	[11]
12		Wang	2022	PDAC	[12]
13	ATRX	Young	2019	PDAC	[13]
14		Li	2018	CRC	[14]
15	DAXX	Tzeng	2006	CRC	[15]
16		Chen	2019	CRC	[16]
17		Liu	2020	CRC	[17]
18		Huang	2022	CRC	[18]
19		Ko	2018	EC	[19]
20		Chen	2020	GC	[20]
21		Wu	2020	GC	[21]
22		Xu	2017	GC	[22]
23	CHAF1A	Zheng	2018	GC	[23]
24		Wang	2019	GC	[24]
25		Xu	2016	HCC	[25]
26	CHAF1B	Peng	2018	HCC	[26]
27	HJURP	Kang	2020	CRC	[27]
28		Chen	2019	HCC	[28]
29		Hu	2017	HCC	[29]
30		Yi	2021	HCC	[30]
31		Chen	2018	HCC	[31]
32		Wang	2020	PDAC	[32]
33	IPO4	Xu	2019	GC	[33]
34	MCM2	Wang	2020	CRC	[34]
35		Giaginis	2009	CRC	[35]
36		Hanna-Morris	2009	CRC	[36]
37		Kato	2003	EC	[37]

ID	Histone chaperone	First author	Year	Tumor	Reference number
38		Huang	2011	EC	[38]
39		Yang	2012	GC	[39]
40		Giaginis	2011	GC	[40]
41		Liu	2013	GC	[41]
42		Tokuyasu	2008	GC	[42]
43		Tang	2022	HCC	[43]
44		Ahmed	2019	HCC	[44]
45	NAP1L1	Queiroz	2020	CRC	[45]
46		Aydin	2020	CRC	[46]
47		Huang	2018	HCC	[47]
48		Chen	2018	HCC	[48]
49		Zhang	2021	HCC	[49]
50		Le	2019	HCC	[50]
		Schimmack	2014	PDAC	[51]
51	NPM1	Wang	2022	CRC	[52]
52		Wong	2013	CRC	[53]
53		Yu	2021	CRC	[54]
54		Grbčić	2021	CRC	[55]
55		Liu	2012	CRC	[56]
56		Nozawa	1996	CRC	[57]
57		Yang	2014	CRC	[58]
58		Tang	2018	CRC	[59]
59		Liu	2022	EC	[60]
60		Leal	2014	GC	[61]
61		Zhou	2016	GC	[62]
62		Zhou	2013	GC	[63]
63		Guo	2022	GC	[64]
64		Li	2014	GC	[65]
65		Lakshmi Ch	2021	GC	[66]
66		Yun	2007	HCC	[67]
67		Xu	2014	HCC	[68]
68		Lo	2013	HCC	[69]
69		Zhang	2022	HCC	[70]
70		Liu	2012	HCC	[71]
71		Li	2017	HCC	[72]
72		Ching	2015	HCC	[73]
73		Zhu	2015	PDAC	[74]
74		Hamilton	2014	PDAC	[75]
75	Nucleolin	Wu	2014	CRC	[76]
76		Reyes-Reyes	2008	CRC	[77]

ID	Histone chaperone	First author	Year	Tumor	Reference number
77		Wu	2020	CRC	[78]
78		Qi	2015	ESCC	[79]
79		Qiu	2013	GC	[80]
80		Devanand	2018	GC	[81]
81		Qiu	2015	HCC	[82]
82		Guo	2014	HCC	[83]
83		Chen	2015	HCC	[84]
84		Burbano De Lara	2021	HCC	[85]
85		Gilles	2016	PDAC	[86]
86		Peng	2010	PDAC	[87]
87		Xu	2019	PDAC	[88]
88	RBAP48	Jin	2018	GC	[89]
89	sNASP	Yu	2017	GC	[90]
90		Kang	2018	HCC	[91]
91	SPT2	Yin	2022	CRC	[92]
92	SPT6	Diao	2021	CRC	[93]
93	SPT16/SSRP1	Shen	2020	HCC	[94]
94	SSRP1	Song	2020	CRC	[95]
95		Wu	2019	CRC	[96]
96		Wang	2019	CRC	[97]
97		Huang	2019	CRC	[98]
98		Jin	2022	GC	[99]
99		Ding	2016	HCC	[100]
100		Min	2021	HCC	[101]
101		Zheng	2020	HCC	[102]
102	TONSL	Wang	2019	GC	[103]
103	VPS72	Chen	2022	HCC	[104]

Abbreviations: ANP32E: Acidic Nuclear Phosphoprotein 32 Family Member E; ASF1A: Anti-Silencing Function 1A; ASF1B: Anti-Silencing Function 1B; ATRX: ATRX Chromatin Remodeler; CHAF1A: Chromatin Assembly Factor 1 Subunit A; CHAF1B: Chromatin Assembly Factor 1 Subunit B; DAXX: Death Domain Associated Protein; HJURP: Holliday Junction Recognition Protein; HIRA; IPO4: Importin 4; MCM2: Minichromosome Maintenance Complex Component 2; NAP1L1: Nucleosome Assembly Protein 1 Like 1; NPM1: Nucleophosmin 1; NR: not reported; NS: not significant; sNASP: Nuclear Autoantigenic Sperm Protein; SPT2: Suppressor Of Ty 2; SPT6: Suppressor Of Ty 6; SPT16: Suppressor Of Ty 16; SSRP1: Structure Specific Recognition Protein 1; TONSL: Tonsoku Like.

Table S3. Clinical events of selected histone chaperones in digestive cancer.

Gene	Tumor	Role	Expression	Correlation with OS	Correlation with DFS	Reference number
ASF1A	HCC	promoter	High	Negative	Negative	[6]
	CRC	promoter	High	Negative	Negative	[4]
ASF1B	HCC	promoter	NR	Negative	Negative	[8]
	HCC	promoter	High	NR	NR	[9]
	GC	promoter	High	Negative	NR	[7]
SPT16	HCC	promoter	High	Negative	Negative	[105]
SSRP1	HCC	promoter	High	Negative	Negative	[105]
	HCC	promoter	High	Negative	Negative	[100]
	GC	promoter	High (RNA)	NR	NR	[99]
	CRC	promoter	High	NR	NR	[95]
	CRC	promoter	High	Negative	Negative	[96]
HJURP	HCC	promoter	High	Negative	NR	[31]
	HCC	promoter	High	NR	NR	[106]
	HCC	promoter	High	Negative	NR	[29]
	HCC	promoter	High	Negative	NR	[107]
	PDAC	promoter	High	NR	NR	[32]
	CRC	promoter	High	Negative	NR	[27]
NPM1	HCC	promoter	High	NR	NR	[108]
	HCC	NR	High	NR	NR	[68]
	HCC	promoter	High	Negative	NR	[109]
	GC	NR	Low	NR	NR	[110]
	GC	promoter	High	Negative	Negative	[62]
	GC	promoter	High	Negative	Negative	[63]
	GC	suppressor	Low	Positive	Positive	[64]
	GC	promoter	High	Negative	Negative	[65]
	PDAC	promoter	High	Negative	NR	[74]
	PDAC	promoter	High (NPM^{S48ph})	NR	NR	[75]
	CRC	promoter	High	NR	NR	[53]
	CRC	promoter	High	Negative	NR	[54]
	CRC	promoter	High	NR	NR	[55]
	CRC	promoter	High	Negative	NR	[56]
	CRC	NR	High	NR	NR	[57]
	CRC	promoter	High	Negative	Negative	[58]
NAP1L1	HCC	promoter	High	Negative	NR	[49]
	HCC	promoter	High	Negative	NR	[50]
	CRC	suppressor	Low	Positive	NR	[45]

Gene	Tumor	Role	Expression	Correlation with OS	Correlation with DFS	Reference number
	CRC	NR	High in serum	NR	NR	[46]
CHAF1A	HCC	promoter	High	Negative	Negative	[25]
	GC	promoter	High	NR	NR	[23]
	GC	promoter	NR	Negative	Negative	[24]
Nucleolin	HCC	promoter	High	Negative	NR	[82]
	HCC	promoter	High	Negative	Negative	[111]
	HCC	promoter	High	Negative	NR	[84]
	GC	dependent on site	High	Dependent on site	NR	[80]
	GC	promoter	High	Negative	NR	[81]
	PDAC	promoter	High	Negative	NR	[86]
	PDAC	dependent on site	High in nucleolus	Positive	NR	[87]
	CRC	promoter	NS	NR	NR	[76]
	ESCC	dependent on site	High	Dependent on site	NR	[79]
DAXX	EC	promoter	High	Negative	NR	[19]
	GC	promoter	High in nucleolus	Negative	Negative	[20]
	GC	suppressor	Low	NR	NR	[21]
	GC	promoter	High in nucleolus	Negative	Negative	[22]
	CRC	NR	Low	NR	NR	[15]
	CRC	suppressor	Low	NR	NR	[16]
	CRC	suppressor	Low (RNA)	NR	NR	[17]
	CRC	promoter	High	NR	NR	[18]
ATRX	CRC	promoter	High (RNA)	NR	NR	[14]
MCM2	HCC	promoter	High	Negative	NR	[43]
	HCC	promoter	High (RNA)	NR	NR	[44]
	EC	promoter	NR	Negative	NR	[37]
	EC	NR	High	NR	NR	[38]
	GC	promoter	High	Negative	Negative	[39]
	GC	NR	High	NS	NR	[40]
	GC	NR	High	Negative	Negative	[41]
	GC	NR	High	Negative	NR	[42]
	CRC	NR	High	NR	NR	[35]

Gene	Tumor	Role	Expression	Correlation with OS	Correlation with DFS	Reference number
	CRC	NR	High	NR	NR	[36]
sNASP	HCC	promoter	High	NR	NR	[91]
IPO4	GC	promoter	High	NR	NR	[33]
SPT2	CRC	promoter	High	Negative	NR	[92]
SPT6	CRC	promoter	High	NR	NR	[93]
ANP32E	PDAC	promoter	High	NR	NR	[3]
	GC	promoter	High (RNA)	NR	NR	[1]

Abbreviations: ANP32E: Acidic Nuclear Phosphoprotein 32 Family Member E; ASF1A: Anti-Silencing Function 1A; ASF1B: Anti-Silencing Function 1B; ATRX: ATRX Chromatin Remodeler; CHAF1A: Chromatin Assembly Factor 1 Subunit A; CHAF1B: Chromatin Assembly Factor 1 Subunit B; DAXX: Death Domain Associated Protein; HJURP: Holliday Junction Recognition Protein; HIRA; IPO4: Importin 4; MCM2: Minichromosome Maintenance Complex Component 2; NAP1L1: Nucleosome Assembly Protein 1 Like 1; NPM1: Nucleophosmin 1; NR: not re-ported; NS: not significant; sNASP: Nuclear Autoantigenic Sperm Protein; SPT2: Suppressor Of Ty 2; SPT6: Suppressor Of Ty 6; SPT16: Suppressor Of Ty 16; SSRP1: Structure Specific Recognition Protein 1; TONSL: Tonsoku Like.

References

1. Zhu, X.; Zou, Y.; Wu, T.; Ni, J.; Tan, Q.; Wang, Q.; Zhang, M. ANP32E contributes to gastric cancer progression via NUF2 upregulation. *Mol Med Rep* **2022**, *26*, doi:10.3892/mmr.2022.12791.
2. Zhang, J.; Lan, Z.; Qiu, G.; Ren, H.; Zhao, Y.; Gu, Z.; Li, Z.; Feng, L.; He, J.; Wang, C. Over-expression of ANP32E is associated with poor prognosis of pancreatic cancer and promotes cell proliferation and migration through regulating β-catenin. *BMC Cancer* **2020**, *20*, 1065, doi:10.1186/s12885-020-07556-z.
3. Ma, Y.S.; Yang, X.L.; Liu, Y.S.; Ding, H.; Wu, J.J.; Shi, Y.; Jia, C.Y.; Lu, G.X.; Zhang, D.D.; Wang, H.M.; et al. Long non-coding RNA NORAD promotes pancreatic cancer stem cell proliferation and self-renewal by blocking microRNA-202-5p-mediated ANP32E inhibition. *J Transl Med* **2021**, *19*, 400, doi:10.1186/s12967-021-03052-5.
4. Liang, X.; Yuan, X.; Yu, J.; Wu, Y.; Li, K.; Sun, C.; Li, S.; Shen, L.; Kong, F.; Jia, J.; et al. Histone Chaperone ASF1A Predicts Poor Outcomes for Patients With Gastrointestinal Cancer and Drives Cancer Progression by Stimulating Transcription of β-Catenin Target Genes. *EBioMedicine* **2017**, *21*, 104-116, doi:10.1016/j.ebiom.2017.06.007.
5. Qiu, F.; Wang, Y.; Chu, X.; Wang, J. ASF1A regulates H4(Y72) phosphorylation and promotes autophagy in colon cancer cells via a kinase activity. *Artif Cells Nanomed Biotechnol* **2019**, *47*, 2754-2763, doi:10.1080/21691401.2019.1617725.
6. Wu, Y.; Li, X.; Yu, J.; Björkholm, M.; Xu, D. ASF1a inhibition induces p53-dependent growth arrest and senescence of cancer cells. *Cell Death Dis* **2019**, *10*, 76, doi:10.1038/s41419-019-1357-z.
7. Chen, C.; Bao, H.; Lin, W.; Chen, X.; Huang, Y.; Wang, H.; Yang, Y.; Liu, J.; Lv, X.; Teng, L. ASF1b is a novel prognostic predictor associated with cell cycle signaling pathway in gastric cancer. *J Cancer* **2022**, *13*, 1985-2000, doi:10.7150/jca.69544.
8. Zhang, S.; Xu, L.; Feng, J.; Tan, D.; Zhu, Y.; Hou, J.; Li, W.; Lv, K.; Wang, W.; Jiang, L.; et al. ASF1B is a Promising Prognostic Biomarker and Correlates With Immunotherapy Efficacy in Hepatocellular Carcinoma. *Front Genet* **2022**, *13*, 842351, doi:10.3389/fgene.2022.842351.
9. Ouyang, X.; Lv, L.; Zhao, Y.; Zhang, F.; Hu, Q.; Li, Z.; Zhu, D.; Li, L. ASF1B Serves as a Potential Therapeutic

Target by Influencing Cell Cycle and Proliferation in Hepatocellular Carcinoma. *Front Oncol* **2021**, *11*, 801506, doi:10.3389/fonc.2021.801506.
10. Zhan, T.; Gao, X.; Wang, G.; Li, F.; Shen, J.; Lu, C.; Xu, L.; Li, Y.; Zhang, J. Construction of Novel lncRNA-miRNA-mRNA Network Associated With Recurrence and Identification of Immune-Related Potential Regulatory Axis in Hepatocellular Carcinoma. *Front Oncol* **2021**, *11*, 626663, doi:10.3389/fonc.2021.626663.
11. Kim, J.H.; Youn, Y.; Lee, J.C.; Kim, J.; Ryu, J.K.; Hwang, J.H. Downregulation of ASF1B inhibits tumor progression and enhances efficacy of cisplatin in pancreatic cancer. *Cancer Biomark* **2022**, *34*, 647-659, doi:10.3233/cbm-210490.
12. Wang, K.; Hao, Z.; Fu, X.; Li, W.; Jiao, A.; Hua, X. Involvement of elevated ASF1B in the poor prognosis and tumorigenesis in pancreatic cancer. *Mol Cell Biochem* **2022**, *477*, 1945-1957, doi:10.1007/s11010-022-04404-5.
13. Young, C.C.; Baker, R.M.; Howlett, C.J.; Hryciw, T.; Herman, J.E.; Higgs, D.; Gibbons, R.; Crawford, H.; Brown, A.; Pin, C.L. The Loss of ATRX Increases Susceptibility to Pancreatic Injury and Oncogenic KRAS in Female But Not Male Mice. *Cell Mol Gastroenterol Hepatol* **2019**, *7*, 93-113, doi:10.1016/j.jcmgh.2018.09.004.
14. Li, X.; Oh, S.; Song, H.; Shin, S.; Zhang, B.; Freeman, W.M.; Janknecht, R. A potential common role of the Jumonji C domain-containing 1A histone demethylase and chromatin remodeler ATRX in promoting colon cancer. *Oncol Lett* **2018**, *16*, 6652-6662, doi:10.3892/ol.2018.9487.
15. Tzeng, S.L.; Cheng, Y.W.; Li, C.H.; Lin, Y.S.; Hsu, H.C.; Kang, J.J. Physiological and functional interactions between Tcf4 and Daxx in colon cancer cells. *J Biol Chem* **2006**, *281*, 15405-15411, doi:10.1074/jbc.M601807200.
16. Chen, Y.C.; Lee, T.H.; Tzeng, S.L. Reduced DAXX Expression Is Associated with Reduced CD24 Expression in Colorectal Cancer. *Cells* **2019**, *8*, 1242, doi:10.3390/cells8101242.
17. Liu, Y.; Guo, F.; Zhu, X.; Guo, W.; Fu, T.; Wang, W. Death Domain-Associated Protein Promotes Colon Cancer Metastasis through Direct Interaction with ZEB1. *J Cancer* **2020**, *11*, 750-758, doi:10.7150/jca.34233.
18. Huang, Y.S.; Wu, C.C.; Chang, C.C.; Huang, S.F.; Kuo, H.Y.; Shih, H.M. Reciprocal regulation of Daxx and PIK3CA promotes colorectal cancer cell growth. *Cell Mol Life Sci* **2022**, *79*, 367, doi:10.1007/s00018-022-04399-8.
19. Ko, T.Y.; Kim, J.I.; Park, E.S.; Mun, J.M.; Park, S.D. The Clinical Implications of Death Domain-Associated Protein (DAXX) Expression. *Korean J Thorac Cardiovasc Surg* **2018**, *51*, 187-194, doi:10.5090/kjtcs.2018.51.3.187.
20. Chen, C.; Sun, X.; Xie, W.; Chen, S.; Hu, Y.; Xing, D.; Xu, J.; Chen, X.; Zhao, Z.; Han, Z.; et al. Opposing biological functions of the cytoplasm and nucleus DAXX modified by SUMO-2/3 in gastric cancer. *Cell Death Dis* **2020**, *11*, 514, doi:10.1038/s41419-020-2718-3.
21. Wu, C.; Ding, H.; Wang, S.; Li, Y.; Liu, S.B.; Wang, X.; Zheng, J.; Xue, T.; Amin, H.M.; Song, Y.H.; et al. DAXX inhibits cancer stemness and epithelial-mesenchymal transition in gastric cancer. *Br J Cancer* **2020**, *122*, 1477-1485, doi:10.1038/s41416-020-0800-3.
22. Xu, J.F.; Zhao, Z.G.; Ye, L.L.; Zhuge, W.; Han, Z.; Zhang, T.M.; Ye, S.S.; Chen, W.J.; Zhu, S.; Shi, L.; et al. Prognostic significance of Daxx NCR (Nuclear/Cytoplasmic Ratio) in gastric cancer. *Cancer Med* **2017**, *6*, 2063-2075, doi:10.1002/cam4.1144.
23. Zheng, L.; Liang, X.; Li, S.; Li, T.; Shang, W.; Ma, L.; Jia, X.; Shao, W.; Sun, P.; Chen, C.; et al. CHAF1A interacts with TCF4 to promote gastric carcinogenesis via upregulation of c-MYC and CCND1 expression. *EBioMedicine* **2018**, *38*, 69-78, doi:10.1016/j.ebiom.2018.11.009.
24. Wang, D.; Li, X.; Shen, B.; Chen, X.; Shu, Y. Histone chaperone CHAF1A impacts the outcome of fluoropyrimidines-based adjuvant therapy in gastric cancer by regulating the expression of thymidylate synthetase. *Gene* **2019**, *716*, 144034, doi:10.1016/j.gene.2019.144034.
25. Xu, M.; Jia, Y.; Liu, Z.; Ding, L.; Tian, R.; Gu, H.; Wang, Y.; Zhang, H.; Tu, K.; Liu, Q. Chromatin assembly factor

1, subunit A (P150) facilitates cell proliferation in human hepatocellular carcinoma. *Onco Targets Ther* **2016**, *9*, 4023-4035, doi:10.2147/ott.S107050.

26. Peng, X.; Fu, H.; Yin, J.; Zhao, Q. CHAF1B knockdown blocks migration in a hepatocellular carcinoma model. *Oncol Rep* **2018**, *40*, 405-413, doi:10.3892/or.2018.6437.
27. Kang, D.H.; Woo, J.; Kim, H.; Kim, S.Y.; Ji, S.; Jaygal, G.; Ahn, T.S.; Kim, H.J.; Kwak, H.J.; Kim, C.J.; et al. Prognostic Relevance of HJURP Expression in Patients with Surgically Resected Colorectal Cancer. *Int J Mol Sci* **2020**, *21*, 7928, doi:10.3390/ijms21217928.
28. Chen, T.; Zhou, Y.; Zhou, W.; Huang, H.; Yin, S.; Xie, H.; Zhou, L.; Zheng, S. HJURP Promotes Epithelial-to-Mesenchymal Transition via Upregulating SPHK1 in Hepatocellular Carcinoma. *International journal of biological sciences* **2019**, *15(6)*, 1139-1147, doi:https://dx.doi.org/10.7150/ijbs.30904.
29. Hu, B.; Wang, Q.; Wang, Y.; Chen, J.; Li, P.; Han, M. Holliday junction-recognizing protein promotes cell proliferation and correlates with unfavorable clinical outcome of hepatocellular carcinoma. *Onco Targets Ther* **2017**, *10*, 2601-2607, doi:10.2147/ott.S127738.
30. Yi, Q.; Liao, X.; Han, C.; Zheng, L.; Li, H.; Yu, Q.; Yan, X.; Chen, X.; Zhu, H.; Zhao, B.; et al. Hypomethylation-driven overexpression of HJURP promotes progression of hepatocellular carcinoma and is associated with poor prognosis. *Biochemical and Biophysical Research Communications* **2021**, *566*, 67-74, doi:https://dx.doi.org/10.1016/j.bbrc.2021.05.102.
31. Chen, T.; Huang, H.; Zhou, Y.; Geng, L.; Shen, T.; Yin, S.; Zhou, L.; Zheng, S. HJURP promotes hepatocellular carcinoma proliferation by destabilizing p21 via the MAPK/ERK1/2 and AKT/GSK3β signaling pathways. *J Exp Clin Cancer Res* **2018**, *37*, 193, doi:10.1186/s13046-018-0866-4.
32. Wang, C.J.; Li, X.; Shi, P.; Ding, H.Y.; Liu, Y.P.; Li, T.; Lin, P.P.; Wang, Y.S.; Zhang, G.Q.; Cao, Y. Holliday junction recognition protein promotes pancreatic cancer growth and metastasis via modulation of the MDM2/p53 signaling. *Cell Death Dis* **2020**, *11*, 386, doi:10.1038/s41419-020-2595-9.
33. Xu, X.; Zhang, X.; Xing, H.; Liu, Z.; Jia, J.; Jin, C.; Zhang, Y. Importin-4 functions as a driving force in human primary gastric cancer. *J Cell Biochem* **2019**, *120*, 12638-12646, doi:10.1002/jcb.28530.
34. Wang, Y.; Chen, H.; Zhang, J.; Cheng, A.S.L.; Yu, J.; To, K.F.; Kang, W. MCM family in gastrointestinal cancer and other malignancies: From functional characterization to clinical implication. *Biochim Biophys Acta Rev Cancer* **2020**, *1874*, 188415, doi:10.1016/j.bbcan.2020.188415.
35. Giaginis, C.; Georgiadou, M.; Dimakopoulou, K.; Tsourouflis, G.; Gatzidou, E.; Kouraklis, G.; Theocharis, S. Clinical significance of MCM-2 and MCM-5 expression in colon cancer: association with clinicopathological parameters and tumor proliferative capacity. *Dig Dis Sci* **2009**, *54*, 282-291, doi:10.1007/s10620-008-0305-z.
36. Hanna-Morris, A.; Badvie, S.; Cohen, P.; McCullough, T.; Andreyev, H.J.; Allen-Mersh, T.G. Minichromosome maintenance protein 2 (MCM2) is a stronger discriminator of increased proliferation in mucosa adjacent to colorectal cancer than Ki-67. *J Clin Pathol* **2009**, *62*, 325-330, doi:10.1136/jcp.2007.054643.
37. Kato, H.; Miyazaki, T.; Fukai, Y.; Nakajima, M.; Sohda, M.; Takita, J.; Masuda, N.; Fukuchi, M.; Manda, R.; Ojima, H.; et al. A new proliferation marker, minichromosome maintenance protein 2, is associated with tumor aggressiveness in esophageal squamous cell carcinoma. *J Surg Oncol* **2003**, *84*, 24-30, doi:10.1002/jso.10287.
38. Huang, B.; Hu, B.; Su, M.; Tian, D.; Guo, Y.; Lian, S.; Liu, Z.; Wu, X.; Li, Q.; Zheng, R.; et al. Potential role of minichromosome maintenance protein 2 as a screening biomarker in esophageal cancer high-risk population in China. *Hum Pathol* **2011**, *42*, 808-816, doi:10.1016/j.humpath.2010.04.022.
39. Yang, C.; Wen, Y.; Li, H.; Zhang, D.; Zhang, N.; Shi, X.; Jiang, B.; Ma, X.; Yang, P.; Tang, H.; et al. Overexpression of minichromosome maintenance 2 predicts poor prognosis in patients with gastric cancer. *Oncol Rep* **2012**, *27*,

135-142, doi:10.3892/or.2011.1473.

40. Giaginis, C.; Giagini, A.; Tsourouflis, G.; Gatzidou, E.; Agapitos, E.; Kouraklis, G.; Theocharis, S. MCM-2 and MCM-5 expression in gastric adenocarcinoma: clinical significance and comparison with Ki-67 proliferative marker. *Dig Dis Sci* **2011**, *56*, 777-785, doi:10.1007/s10620-010-1348-5.
41. Liu, M.; Li, J.S.; Tian, D.P.; Huang, B.; Rosqvist, S.; Su, M. MCM2 expression levels predict diagnosis and prognosis in gastric cardiac cancer. *Histol Histopathol* **2013**, *28*, 481-492, doi:10.14670/hh-28.481.
42. Tokuyasu, N.; Shomori, K.; Nishihara, K.; Kawaguchi, H.; Fujioka, S.; Yamaga, K.; Ikeguchi, M.; Ito, H. Minichromosome maintenance 2 (MCM2) immunoreactivity in stage III human gastric carcinoma: clinicopathological significance. *Gastric Cancer* **2008**, *11*, 37-46, doi:10.1007/s10120-008-0451-1.
43. Tang, Z.; Yang, Y.; Chen, W.; Li, E.; Liang, T. Demethylation at enhancer upregulates MCM2 and NUP37 expression predicting poor survival in hepatocellular carcinoma patients. *J Transl Med* **2022**, *20*, 49, doi:10.1186/s12967-022-03249-2.
44. Ahmed, M.Y.; Salah, M.M.; Kassim, S.K.; Abdelaal, A.; Elayat, W.M.; Mohamed, D.A.; Fouly, A.E.; Abu-Zahra, F.A.E. Evaluation of the diagnostic and therapeutic roles of non-coding RNA and cell proliferation related gene association in hepatocellular carcinoma. *Gene* **2019**, *706*, 97-105, doi:10.1016/j.gene.2019.04.054.
45. Queiroz, C.J.S.; Song, F.; Reed, K.R.; Al-Khafaji, N.; Clarke, A.R.; Vimalachandran, D.; Miyajima, F.; Pritchard, D.M.; Jenkins, J.R. NAP1L1: A Novel Human Colorectal Cancer Biomarker Derived From Animal Models of Apc Inactivation. *Front Oncol* **2020**, *10*, 1565, doi:10.3389/fonc.2020.01565.
46. Aydin, M.A.; Gul, G.; Kiziltan, R.; Algul, S.; Kemik, O. Nucleosome assembly protein 1-like 1 (NAP1L1) in colon cancer patients: a potential biomarker with diagnostic and prognostic utility. *Eur Rev Med Pharmacol Sci* **2020**, *24*, 10512-10517, doi:10.26355/eurrev_202010_23403.
47. Huang, Y.; Xiang, B.; Liu, Y.; Wang, Y.; Kan, H. LncRNA CDKN2B-AS1 promotes tumor growth and metastasis of human hepatocellular carcinoma by targeting let-7c-5p/NAP1L1 axis. *Cancer Lett* **2018**, *437*, 56-66, doi:10.1016/j.canlet.2018.08.024.
48. Chen, Z.; Gao, W.; Pu, L.; Zhang, L.; Han, G.; Zuo, X.; Zhang, Y.; Li, X.; Shen, H.; Wu, J.; et al. PRDM8 exhibits antitumor activities toward hepatocellular carcinoma by targeting NAP1L1. *Hepatology* **2018**, *68*, 994-1009, doi:10.1002/hep.29890.
49. Zhang, Y.W.; Chen, Q.; Li, B.; Li, H.Y.; Zhao, X.K.; Xiao, Y.Y.; Liu, S.; Zuo, S. NAP1L1 Functions as a Tumor Promoter via Recruiting Hepatoma-Derived Growth Factor/c-Jun Signal in Hepatocellular Carcinoma. *Front Cell Dev Biol* **2021**, *9*, 659680, doi:10.3389/fcell.2021.659680.
50. Le, Y.; Kan, A.; Li, Q.J.; He, M.K.; Chen, H.L.; Shi, M. NAP1L1 is a prognostic biomarker and contribute to doxorubicin chemotherapy resistance in human hepatocellular carcinoma. *Cancer Cell Int* **2019**, *19*, 228, doi:10.1186/s12935-019-0949-0.
51. Schimmack, S.; Taylor, A.; Lawrence, B.; Alaimo, D.; Schmitz-Winnenthal, H.; Büchler, M.W.; Modlin, I.M.; Kidd, M. A mechanistic role for the chromatin modulator, NAP1L1, in pancreatic neuroendocrine neoplasm proliferation and metastases. *Epigenetics Chromatin* **2014**, *7*, 15, doi:10.1186/1756-8935-7-15.
52. Wang, D.; Li, Y.; Liu, Y.; Cheng, S.; Liu, F.; Zuo, R.; Ding, C.; Shi, S.; Liu, G. NPM1 promotes cell proliferation by targeting PRDX6 in colorectal cancer. *Int J Biochem Cell Biol* **2022**, *147*, 106233, doi:10.1016/j.biocel.2022.106233.
53. Wong, J.C.; Hasan, M.R.; Rahman, M.; Yu, A.C.; Chan, S.K.; Schaeffer, D.F.; Kennecke, H.F.; Lim, H.J.; Owen, D.; Tai, I.T. Nucleophosmin 1, upregulated in adenomas and cancers of the colon, inhibits p53-mediated cellular senescence. *Int J Cancer* **2013**, *133*, 1567-1577, doi:10.1002/ijc.28180.
54. Yu, A.C.Y.; Chern, Y.J.; Zhang, P.; Pasiliao, C.C.; Rahman, M.; Chang, G.; Ren, J.; Tai, I.T. Inhibition of

nucleophosmin 1 suppresses colorectal cancer tumor growth of patient -derived xenografts via activation of p53 and inhibition of AKT. *Cancer Biol Ther* **2021**, *22*, 112-123, doi:10.1080/15384047.2020.1839278.
55. Grbčić, P.; Fučkar Čupić, D.; Gamberi, T.; Kraljević Pavelić, S.; Sedić, M. Proteomic Profiling of BRAFV600E Mutant Colon Cancer Cells Reveals the Involvement of Nucleophosmin/c-Myc Axis in Modulating the Response and Resistance to BRAF Inhibition by Vemurafenib. *Int J Mol Sci* **2021**, *22*, doi:10.3390/ijms22126174.
56. Liu, Y.; Zhang, F.; Zhang, X.F.; Qi, L.S.; Yang, L.; Guo, H.; Zhang, N. Expression of nucleophosmin/NPM1 correlates with migration and invasiveness of colon cancer cells. *J Biomed Sci* **2012**, *19*, 53, doi:10.1186/1423-0127-19-53.
57. Nozawa, Y.; Van Belzen, N.; Van der Made, A.C.; Dinjens, W.N.; Bosman, F.T. Expression of nucleophosmin/B23 in normal and neoplastic colorectal mucosa. *J Pathol* **1996**, *178*, 48-52, doi:10.1002/(sici)1096-9896(199601)178:1<48::Aid-path432>3.0.Co;2-y.
58. Yang, Y.F.; Zhang, X.Y.; Yang, M.; He, Z.H.; Peng, N.F.; Xie, S.R.; Xie, Y.F. Prognostic role of nucleophosmin in colorectal carcinomas. *Asian Pac J Cancer Prev* **2014**, *15*, 2021-2026, doi:10.7314/apjcp.2014.15.5.2021.
59. Tang, J.; Chen, H.; Wong, C.C.; Liu, D.; Li, T.; Wang, X.; Ji, J.; Sung, J.J.; Fang, J.Y.; Yu, J. DEAD-box helicase 27 promotes colorectal cancer growth and metastasis and predicts poor survival in CRC patients. *Oncogene* **2018**, *37*, 3006-3021, doi:10.1038/s41388-018-0196-1.
60. Liu, Y.; Li, C.; Fang, L.; Wang, L.; Liu, H.; Tian, H.; Zheng, Y.; Fan, T.; He, J. Lipid metabolism-related lncRNA SLC25A21-AS1 promotes the progression of oesophageal squamous cell carcinoma by regulating the NPM1/c-Myc axis and SLC25A21 expression. *Clin Transl Med* **2022**, *12*, e944, doi:10.1002/ctm2.944.
61. Leal, M.F.; Mazzotti, T.K.F.; Calcagno, D.Q.; Cirilo, P.D.R.; Martinez, M.C.; Demachki, S.; Assumpcao, P.P.; Chammas, R.; Burbano, R.R.; Smith, M.C. Deregulated expression of Nucleophosmin 1 in gastric cancer and its clinicopathological implications. *BMC Gastroenterology* **2014**, *14(1) (no pagination)*, doi:https://dx.doi.org/10.1186/1471-230X-14-9.
62. Zhou, F.; Chen, E.; You, D.; Song, Y.; Sun, Z.; Yue, L. Both high expression of nucleophosmin/B23 and CRM1 predicts poorer prognosis in human gastric cancer. *Apmis* **2016**, *124*, 1046-1053, doi:10.1111/apm.12604.
63. Zhou, F.; Qiu, W.; Sun, L.; Xiang, J.; Sun, X.; Sui, A.; Ding, A.; Yue, L. Clinical significance of nucleophosmin/B23 and human epidermal growth factor receptor 2/neu expressions in gastric cancers. *Apmis* **2013**, *121*, 582-591, doi:10.1111/apm.12043.
64. Guo, C.A.; Su, X.L.; Wang, W.J.; Xia, T.H.; Cao, X.M.; Yuan, S.B.; Wang, W.A.; Zhang, A.; Liu, H.B. NPM1 is a diagnostic and prognostic biomarker associated with the clinicopathological characteristics of gastric cancer. *Neoplasma.* **2022**, *20*, 965-975, doi:https://dx.doi.org/10.4149/neo_2022_220303N237.
65. Li, Y.; Sun, Z.; Liu, K.; Qiu, W.; Yao, R.; Feng, T.; Xin, C.; Yue, L. Prognostic significance of the co-expression of nucleophosmin and trefoil factor 3 in postoperative gastric cancer patients. *Molecular and Clinical Oncology* **2014**, *2*, 1055-1061, doi:https://dx.doi.org/10.3892/mco.2014.351.
66. Lakshmi Ch, N.P.; Sivagnanam, A.; Raja, S.; Mahalingam, S. Molecular basis for RASSF10/NPM/RNF2 feedback cascade-mediated regulation of gastric cancer cell proliferation. *J Biol Chem* **2021**, *297*, 100935, doi:10.1016/j.jbc.2021.100935.
67. Yun, J.P.; Miao, J.; Chen, G.G.; Tian, Q.H.; Zhang, C.Q.; Xiang, J.; Fu, J.; Lai, P.B.S. Increased expression of nucleophosmin/B23 in hepatocellular carcinoma and correlation with clinicopathological parameters. *British Journal of Cancer* **2007**, *96(3)*, 477-484, doi:https://dx.doi.org/10.1038/sj.bjc.6603574.
68. Xu, D.H.; Liu, F.; Li, X.; Chen, X.F.; Jing, G.J.; Wu, F.Y.; Shi, S.L.; Li, Q.F. Regulatory role of nucleophosmin during the differentiation of human liver cancer cells. *Int J Oncol* **2014**, *45*, 264-272, doi:10.3892/ijo.2014.2407.

69. Lo, S.J.; Fan, L.C.; Tsai, Y.F.; Lin, K.Y.; Huang, H.L.; Wang, T.H.; Liu, H.; Chen, T.C.; Huang, S.F.; Chang, C.J.; et al. A novel interaction of nucleophosmin with BCL2-associated X protein regulating death evasion and drug sensitivity in human hepatoma cells. *Hepatology* **2013**, *57(5)*, 1893-1905, doi:https://dx.doi.org/10.1002/hep.26209.
70. Zhang, D.; Wu, F.; Song, J.; Meng, M.; Fan, X.; Lu, C.; Weng, Q.; Fang, S.; Zheng, L.; Tang, B.; et al. A role for the NPM1/PTPN14/YAP axis in mediating hypoxia-induced chemoresistance to sorafenib in hepatocellular carcinoma. *Cancer Cell Int* **2022**, *22*, 65, doi:10.1186/s12935-022-02479-0.
71. Liu, D.; Qian, D.; Dai, J.; An, Y.; Jiang, S.; Stanley, B.; Yang, J.; Wang, B.; Liu, X.; Liu, D.X. Nucleophosmin (NPM1/B23) interacts with activating transcription factor 5 (ATF5) protein and promotes proteasome- and caspase-dependent ATF5 degradation in hepatocellular carcinoma cells. *Journal of Biological Chemistry* **2012**, *287(23)*, 19599-19609, doi:https://dx.doi.org/10.1074/jbc.M112.363622.
72. Li, X.; Xu, D.H.; Liu, F.; Liu, G.Y.; Lu, K.; Deng, X.L.; Li, Q.F.; Shi, S.L. Relocation of NPM Affects the Malignant Phenotypes of Hepatoma SMMC-7721 Cells. *J Cell Biochem* **2017**, *118*, 3225-3236, doi:10.1002/jcb.25971.
73. Ching, R.H.H.; Lau, E.Y.T.; Ling, P.M.T.; Lee, J.M.F.; Ma, M.K.F.; Cheng, B.Y.L.; Lo, R.C.L.; Ng, I.O.L.; Lee, T.K.W. Phosphorylation of Nucleophosmin at Threonine 234/237 is associated with HCC metastasis. *Oncotarget* **2015**, *6*, 43483-43495, doi:https://dx.doi.org/10.18632/oncotarget.5820.
74. Zhu, Y.; Shi, M.; Chen, H.; Gu, J.; Zhang, J.; Shen, B.; Deng, X.; Xie, J.; Zhan, X.; Peng, C. NPM1 activates metabolic changes by inhibiting FBP1 while promoting the tumorigenicity of pancreatic cancer cells. *Oncotarget* **2015**, *6*, 21443-21451, doi:10.18632/oncotarget.4167.
75. Hamilton, G.; Abraham, A.G.; Morton, J.; Sampson, O.; Pefani, D.E.; Khoronenkova, S.; Grawenda, A.; Papaspyropoulos, A.; Jamieson, N.; McKay, C.; et al. AKT regulates NPM dependent ARF localization and p53mut stability in tumors. *Oncotarget* **2014**, *5*, 6142-6167, doi:10.18632/oncotarget.2178.
76. Wu, D.M.; Zhang, P.; Liu, R.Y.; Sang, Y.X.; Zhou, C.; Xu, G.C.; Yang, J.L.; Tong, A.P.; Wang, C.T. Phosphorylation and changes in the distribution of nucleolin promote tumor metastasis via the PI3K/Akt pathway in colorectal carcinoma. *FEBS Lett* **2014**, *588*, 1921-1929, doi:10.1016/j.febslet.2014.03.047.
77. Reyes-Reyes, E.M.; Akiyama, S.K. Cell-surface nucleolin is a signal transducing P-selectin binding protein for human colon carcinoma cells. *Exp Cell Res* **2008**, *314*, 2212-2223, doi:10.1016/j.yexcr.2008.03.016.
78. Wu, R.; Li, L.; Bai, Y.; Yu, B.; Xie, C.; Wu, H.; Zhang, Y.; Huang, L.; Yan, Y.; Li, X.; et al. The long noncoding RNA LUCAT1 promotes colorectal cancer cell proliferation by antagonizing Nucleolin to regulate MYC expression. *Cell Death Dis* **2020**, *11*, 908, doi:10.1038/s41419-020-03095-4.
79. Qi, J.; Li, H.; Liu, N.; Xing, Y.; Zhou, G.; Wu, Y.; Liu, Y.; Chen, W.; Yue, J.; Han, B.; et al. The implications and mechanisms of the extra-nuclear nucleolin in the esophageal squamous cell carcinomas. *Med Oncol* **2015**, *32*, 45, doi:10.1007/s12032-015-0484-3.
80. Qiu, W.; Zhou, F.; Zhang, Q.; Sun, X.; Shi, X.; Liang, Y.; Wang, X.; Yue, L. Overexpression of nucleolin and different expression sites both related to the prognosis of gastric cancer. *Apmis* **2013**, *121*, 919-925, doi:10.1111/apm.12131.
81. Devanand, P.; Oya, Y.; Sundaramoorthy, S.; Song, K.Y.; Watanabe, T.; Kobayashi, Y.; Shimizu, Y.; Hong, S.A.; Suganuma, M.; Lim, I.K. Inhibition of TNFα-interacting protein α (Tipα)-associated gastric carcinogenesis by BTG2(/TIS21) via downregulating cytoplasmic nucleolin expression. *Exp Mol Med* **2018**, *50*, e449, doi:10.1038/emm.2017.281.
82. Qiu, W.; Wang, G.; Sun, X.; Ye, J.; Wei, F.; Shi, X.; Lv, G. The involvement of cell surface nucleolin in the initiation of CCR6 signaling in human hepatocellular carcinoma. *Med Oncol* **2015**, *32*, 75, doi:10.1007/s12032-015-0530-1.
83. Guo, X.; Yu, L.; Dong, J.; Li, R.; Li, B.; Liu, H.; Yang, M.; Zhao, J. Increased level of nucleolin confers to aggressive

tumor progression and poor prognosis in patients with hepatocellular carcinoma after hepatectomy. *Hepatology* **2014**, *1)*, 856A, doi:https://dx.doi.org/10.1002/hep.27524.

84. Chen, S.C.; Hu, T.H.; Huang, C.C.; Kung, M.L.; Chu, T.H.; Yi, L.N.; Huang, S.T.; Chan, H.H.; Chuang, J.H.; Liu, L.F.; et al. Hepatoma-derived growth factor/nucleolin axis as a novel oncogenic pathway in liver carcinogenesis. *Oncotarget* **2015**, *6*, 16253-16270, doi:10.18632/oncotarget.3608.
85. Burbano De Lara, S.; Tran, D.D.H.; Allister, A.B.; Polenkowski, M.; Nashan, B.; Koch, M.; Tamura, T. C20orf204, a hepatocellular carcinoma-specific protein interacts with nucleolin and promotes cell proliferation. *Oncogenesis* **2021**, *10*, 31, doi:https://dx.doi.org/10.1038/s41389-021-00320-3.
86. Gilles, M.E.; Maione, F.; Cossutta, M.; Carpentier, G.; Caruana, L.; Di Maria, S.; Houppe, C.; Destouches, D.; Shchors, K.; Prochasson, C.; et al. Nucleolin Targeting Impairs the Progression of Pancreatic Cancer and Promotes the Normalization of Tumor Vasculature. *Cancer Res* **2016**, *76*, 7181-7193, doi:10.1158/0008-5472.Can-16-0300.
87. Peng, L.; Liang, J.; Wang, H.; Song, X.; Rashid, A.; Gomez, H.F.; Corley, L.J.; Abbruzzese, J.L.; Fleming, J.B.; Evans, D.B.; et al. High levels of nucleolar expression of nucleolin are associated with better prognosis in patients with stage II pancreatic ductal adenocarcinoma. *Clin Cancer Res* **2010**, *16*, 3734-3742, doi:10.1158/1078-0432.Ccr-09-3411.
88. Xu, C.; Wang, Y.; Tu, Q.; Zhang, Z.; Chen, M.; Mwangi, J.; Li, Y.; Jin, Y.; Zhao, X.; Lai, R. Targeting surface nucleolin induces autophagy-dependent cell death in pancreatic cancer via AMPK activation. *Oncogene* **2019**, *38*, 1832-1844, doi:10.1038/s41388-018-0556-x.
89. Jin, X.; Jiang, R.; Xiang, Y.; Fan, Z.; Wu, Z.; Yang, B.; Yang, L.; Wei, S.; Yang, Y. Overexpression of retinoblastoma-binding protein 4 contributes to the radiosensitivity of AGS gastric cancer cells via phosphoinositide3-kinase/protein kinase B pathway suppression. *Mol Med Rep* **2018**, *18*, 1571-1581, doi:10.3892/mmr.2018.9153.
90. Yu, B.; Chen, X.; Li, J.; Gu, Q.; Zhu, Z.; Li, C.; Su, L.; Liu, B. microRNA-29c inhibits cell proliferation by targeting NASP in human gastric cancer. *BMC Cancer* **2017**, *17*, 109, doi:10.1186/s12885-017-3096-9.
91. Kang, X.; Feng, Y.; Gan, Z.; Zeng, S.; Guo, X.; Chen, X.; Zhang, Y.; Wang, C.; Liu, K.; Chen, X.; et al. NASP antagonize chromatin accessibility through maintaining histone H3K9me1 in hepatocellular carcinoma. *Biochim Biophys Acta Mol Basis Dis* **2018**, *1864*, 3438-3448, doi:10.1016/j.bbadis.2018.07.033.
92. Yin, L.; Tang, Y.; Xiao, M.; Li, M.; Huang Fu, Z.M.; Wang, Y.L. The role of histone chaperone spty2d1 in human colorectal cancer. *Mol Cell Probes* **2022**, *64*, 101832, doi:10.1016/j.mcp.2022.101832.
93. Diao, C.; Guo, P.; Yang, W.; Sun, Y.; Liao, Y.; Yan, Y.; Zhao, A.; Cai, X.; Hao, J.; Hu, S.; et al. SPT6 recruits SND1 to co-activate human telomerase reverse transcriptase to promote colon cancer progression. *Mol Oncol* **2021**, *15*, 1180-1202, doi:10.1002/1878-0261.12878.
94. Shen, J.; Chen, M.; Lee, D.; Law, C.T.; Wei, L.; Tsang, F.H.C.; Chin, D.W.C.; Cheng, C.L.H.; Lee, J.M.F.; Ng, I.O.L.; et al. Histone chaperone FACT complex mediates oxidative stress response to promote liver cancer progression. *Gut* **2020**, *69(2)*, 329-342, doi:https://dx.doi.org/10.1136/gutjnl-2019-318668.
95. Song, H.; Zeng, J.; Roychoudhury, S.; Biswas, P.; Mohapatra, B.; Ray, S.; Dowlatshahi, K.; Wang, J.; Band, V.; Talmon, G.; et al. Targeting Histone Chaperone FACT Complex Overcomes 5-Fluorouracil Resistance in Colon Cancer. *Mol Cancer Ther* **2020**, *19*, 258-269, doi:10.1158/1535-7163.Mct-19-0600.
96. Wu, W.; He, K.; Guo, Q.; Chen, J.; Zhang, M.; Huang, K.; Yang, D.; Wu, L.; Deng, Y.; Luo, X.; et al. SSRP1 promotes colorectal cancer progression and is negatively regulated by miR-28-5p. *J Cell Mol Med* **2019**, *23*, 3118-3129, doi:10.1111/jcmm.14134.

97. Wang, Q.; Jia, S.; Jiao, Y.; Xu, L.; Wang, D.; Chen, X.; Hu, X.; Liang, H.; Wen, N.; Zhang, S.; et al. SSRP1 influences colorectal cancer cell growth and apoptosis via the AKT pathway. *Int J Med Sci* **2019**, *16*, 1573-1582, doi:10.7150/ijms.38439.
98. Huang, H.; Cai, L.; Li, R.; Ye, L.; Chen, Z. A novel lncRNA LOC101927746 accelerates progression of colorectal cancer via inhibiting miR-584-3p and activating SSRP1. *Biochem Biophys Res Commun* **2019**, *509*, 734-738, doi:10.1016/j.bbrc.2018.12.174.
99. Jin, G.; Zhao, R.; Zhang, J.; Cao, T.; Tang, T. SSRP1 affects the growth and apoptosis of gastric cancer cells through AKT pathway. *J Med Biochem* **2022**, *41*, 100-107, doi:10.5937/jomb0-33374.
100. Ding, Q.; He, K.; Luo, T.; Deng, Y.; Wang, H.; Liu, H.; Zhang, J.; Chen, K.; Xiao, J.; Duan, X.; et al. SSRP1 Contributes to the Malignancy of Hepatocellular Carcinoma and Is Negatively Regulated by miR-497. *Mol Ther* **2016**, *24*, 903-914, doi:10.1038/mt.2016.9.
101. Min, J.; Jin, D.; Zhang, F.; Kang, Y.; Qi, Y.; Du, P. DLG1-AS1 is activated by MYC and drives the proliferation and migration of hepatocellular carcinoma cells through miR-497-5p/SSRP1 axis. *Cancer Cell International* **2021**, *21*, 16, doi:https://dx.doi.org/10.1186/s12935-020-01667-0.
102. Zheng, S.; Guo, Y.; Dai, L.; Liang, Z.; Yang, Q.; Yi, S. Long intergenic noncoding RNA01134 accelerates hepatocellular carcinoma progression by sponging microRNA-4784 and downregulating structure specific recognition protein 1. *Bioengineered* **2020**, *11*, 1016-1026, doi:10.1080/21655979.2020.1818508.
103. Wang, P.; Yang, X.; Zhao, L.; Liu, D.; Liu, J.; Ding, Y. A novel long non-coding RNA TONSL-AS1 regulates progression of gastric cancer via activating TONSL. *Exp Cell Res* **2019**, *382*, 111453, doi:10.1016/j.yexcr.2019.05.034.
104. Chen, T.; Tu, Y.; Lv, D.; Lin, K.; Tang, H.; Huang, W. Vacuolar protein sorting-associated protein 72 homolog (VPS72) binding to lysine acetyltransferase 5 (KAT5) promotes the proliferation, invasion and migration of hepatocellular carcinoma through regulating phosphatidylinositol 3-kinase (PI3K)/protein kinase B (AKT) signaling pathway. *Bioengineered* **2022**, *13*, 9197-9210, doi:10.1080/21655979.2022.2056692.
105. Shen, J.; Chen, M.; Lee, D.; Law, C.T.; Wei, L.; Tsang, F.H.; Chin, D.W.; Cheng, C.L.; Lee, J.M.; Ng, I.O.; et al. Histone chaperone FACT complex mediates oxidative stress response to promote liver cancer progression. *Gut* **2020**, *69*, 329-342, doi:10.1136/gutjnl-2019-318668.
106. Chen, T.; Zhou, L.; Zhou, Y.; Zhou, W.; Huang, H.; Yin, S.; Xie, H.; Zhou, L.; Zheng, S. HJURP Promotes Epithelial-to-Mesenchymal Transition via Upregulating SPHK1 in Hepatocellular Carcinoma. *Int J Biol Sci* **2019**, *15*, 1139-1147, doi:10.7150/ijbs.30904.
107. Li, Y.; Yi, Q.; Liao, X.; Han, C.; Zheng, L.; Li, H.; Yu, Q.; Yan, X.; Chen, X.; Zhu, H.; et al. Hypomethylation-driven overexpression of HJURP promotes progression of hepatocellular carcinoma and is associated with poor prognosis. *Biochem Biophys Res Commun* **2021**, *566*, 67-74, doi:10.1016/j.bbrc.2021.05.102.
108. Yun, J.P.; Miao, J.; Chen, G.G.; Tian, Q.H.; Zhang, C.Q.; Xiang, J.; Fu, J.; Lai, P.B. Increased expression of nucleophosmin/B23 in hepatocellular carcinoma and correlation with clinicopathological parameters. *Br J Cancer* **2007**, *96*, 477-484, doi:10.1038/sj.bjc.6603574.
109. Lo, S.J.; Fan, L.C.; Tsai, Y.F.; Lin, K.Y.; Huang, H.L.; Wang, T.H.; Liu, H.; Chen, T.C.; Huang, S.F.; Chang, C.J.; et al. A novel interaction of nucleophosmin with BCL2-associated X protein regulating death evasion and drug sensitivity in human hepatoma cells. *Hepatology* **2013**, *57*, 1893-1905, doi:10.1002/hep.26209.
110. Leal, M.F.; Mazzotti, T.K.; Calcagno, D.Q.; Cirilo, P.D.; Martinez, M.C.; Demachki, S.; Assumpção, P.P.; Chammas, R.; Burbano, R.R.; Smith, M.C. Deregulated expression of Nucleophosmin 1 in gastric cancer and its clinicopathological implications. *BMC Gastroenterol* **2014**, *14*, 9, doi:10.1186/1471-230x-14-9.

111. Guo, X.; Xiong, L.; Yu, L.; Li, R.; Wang, Z.; Ren, B.; Dong, J.; Li, B.; Wang, D. Increased level of nucleolin confers to aggressive tumor progression and poor prognosis in patients with hepatocellular carcinoma after hepatectomy. *Diagn Pathol* **2014**, *9*, 175, doi:10.1186/s13000-014-0175-y.
